# Dynamic allostery: a novel mechanism regulating autocrine and paracrine TGF-β signalling

**DOI:** 10.1038/s41392-024-02099-2

**Published:** 2025-01-03

**Authors:** Hendrik Ungefroren, Jens Uwe Marquardt

**Affiliations:** 1 First Department of Medicine, University Hospital Schleswig-Holstein (UKSH), Campus Lübeck, Lübeck, Germany; 2 Clinic of Surgery, University Hospital Schleswig-Holstein (UKSH), Campus Lübeck, Lübeck, Germany; 3 Institute of Pathology, University Hospital Schleswig-Holstein (UKSH), Campus Kiel, Kiel, Germany

**Keywords:** Cell biology, Oncology

In a recent landmark study published in *Cell*^1^ Jin and colleagues convincingly demonstrated that mature transforming growth factor-β1 (mTGF-β1) can be activated without release from its latent form (L-TGF-β), and that binding of unreleased mTGF-β to its receptors induces autocrine signalling rather than the conventional paracrine effects. These findings contradict the current dogma that mTGF-β1 requires physical dissociation and release from L-TGF-β1 in order to be able to bind to the TGF-β receptors (TGF-βRs) and signal.

**Fig. 1:**
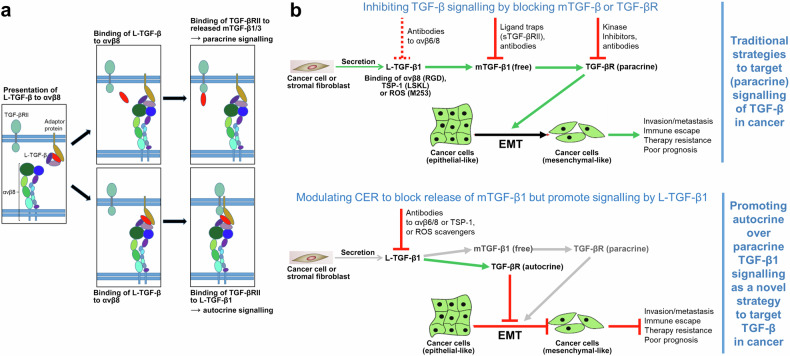
Cartoons to illustrate the model of L-TGF-β activation and signalling without release of mTGF-β, and modes of targeting TGF-β activation through modulation of CER to block EMT. **a** Activation of L-TGF-β for either paracrine or autocrine signalling proceeds in three steps: (i) initially, integrin αvβ8 surveys the environment for cells presenting L-TGF-β on the cell surface together with an adaptor protein, i.e., GARP (left-hand panel, brownish oval); (ii) binding of one cell expressing αvβ8 to another cell expressing L-TGF-β increases CER in L-TGF-β, resulting either in release of mTGF-β (top middle panel, red oval), or in exposure of the active domain of the mTGF-β homodimer while remaining in the L-TGF-β-GARP-αvβ8 complex (bottom middle panel); (iii) released freely diffusible mTGF-β1 (or 3) is likely to bind to TGF-βRII expressed by neighbouring cells (top right-hand panel) to trigger pTGF-β signalling, while the exposed active domain of mTGF-β within L-TGF-β1 can bind to TGF-βRII expressed by the same cell to induce autocrine signalling (bottom right-hand panel). **b** Modes of targeting the balance of paracrine to autocrine TGF-β1 production to interfere with EMT and carcinoma progression. Upper panel (showing the traditional strategies to block pTGF-β1 signalling in cancer): L-TGF-β1 synthesised and secreted by cancer cells or tumour stromal fibroblasts (top left) is activated by αvβ8 binding and the released mTGF-β bound by TGF-βRs on neighbouring cancer cells to initiate signalling and promote EMT. EMT favours invasion/metastasis, an immunosuppressive TME, resistance to various types of anticancer therapies and reduces patient survival.^5^ In immuno-oncology, the binary dogmatic concept of TGF-β activation (either latent or active) has led to therapeutic approaches targeting released mTGF-β1 by antibodies or ligand traps, i.e., soluble TGF-βRII (sTGF-βRII), or antibodies to or kinase inhibitors of TGF-βRI/II, while inhibitors of L-TGF-β1 activation, i.e., antibodies to αvβ6 or αvβ8, are only beginning to be used^2^ (indicated by the stippled red lines). However, many of these clinical trials have either failed due to lack of efficacy or poor activity of available antibodies^2^ or patients experienced unwanted or even harmful side effects when physiological benign functions of TGF-β1 were compromised. To avoid these problems a more selective and localised targeting of TGF-β1 signalling is needed, e.g., of its activation process in the TME to eventually convert TGF-β‘s effects from pro- to antitumourigenic. To this end, activation by the matrix protein TSP-1 (via binding to LSKL in LAP) or ROS (via oxidative modification/M253 in LAP) may be targeted for inhibition, which is expected to also reduce dynamic allostery since all these activators of L-TGF-β1 were either proven (αvβ8) or suspected (TSP-1, ROS) to induce CER. Lower panel (showing the potential role of CER in promoting aTGF-β1 signalling in cancer): as outlined in the main text, signalling by aTGF-β1 can function in an antagonistic manner to the paracrine action of released mTGF-β1 (the function of which can be mimicked in vitro by exogenously applied TGF-β1), suggesting that promoting aTGF-β over pTGF-β can exert antitumour effects.^3^ Therapeutically modulating CER in L-TGF-β1 by blocking its activation and hence release of mTGF-β1, while at the same time promoting autocrine signalling by L-TGF-β1 may represent a novel and innovative approach to interfere with TGF-β1-induced EMT and its protumourigenic consequences. This requires adjusting CER to an extent that is insufficient to induce release of mTGF-β1, but sufficient to expose it to TGF-βRII. This may be achievable with blocking antibodies to αvβ8 or TSP-1, or ROS scavengers. CER conformational entropy redistribution, EMT epithelial–mesenchymal transition. Green arrows denote stimulatory and red straight lines inhibitory interactions. The green-coloured cells represent cancer cells

The three isoforms of TGF-β (1–3) are key regulators of tissue homoeostasis, differentiation and growth. Malfunction of their signalling is implicated in many pathologies, including fibrosis and cancer. While TGF-β1 is primarily involved in controlling immune responses postnatally, TGF-β2 and 3 exert effects during embryonic development. Production of TGF-βs is tightly regulated and occurs, to a large extent, at a post-translational level. Nearly all cells produce TGF-β in a latent, inactive form (L-TGF-β) in which mTGF-β is non-covalently bound to its pro-domain in a complex termed “latent-associated peptide” (LAP). In immune cells, L-TGF-β is presented in association with the membrane-bound adaptor protein GARP (L-TGF-β-GARP) on the cell surface (Fig. 1a). Following binding of TGF-β1 or 3 LAPs to integrin avβ6 or avβ8, pro-TGF-β dimer is eventually cleaved into LAP and the mTGF-β ligand, followed by release and diffusion of the latter from the site of “activation” (Fig. 1a). In epithelial cells, L-TGF-β may be complexed with another, secreted adaptor protein, LTBP (latent TGF-β binding protein) and stored as L-TGF-β-LTBP in the extracellular matrix (ECM). Since LTBP is connected to rigid ECM, binding of integrin creates mechanical forces (countertraction) that deform LAP and facilitate release of mTGF-β.^2^ Activation by either avβ6 or avβ8 is crucial for almost all functions of TGF-β1 and some of TGF-β3.

By employing highly sophisticated methods and genetic mouse models, the authors contrast the current dogma and provide evidence that TGF-β1 can also induce signalling in a non-activated form, i.e., while still residing in a L-TGF-β-GARP-avβ8 complex (Fig. 1a). Herein, binding to avβ8 alters intrinsic flexibility of L-TGF-β1 to expose TGF-β1 to nearby receptors expressed by the same cell, hence resulting in autocrine TGF-β (aTGF-β) signalling. This scenario is a prominent example of allosteric regulation of protein function,^1^ a mechanism where ligand binding at a distal site of the protein affects binding affinity (or catalytic activity) at the active site. This process traditionally involves transition through discrete conformational states resulting from folding/unfolding of its domains. However, an allosteric ligand may also induce changes of dynamic properties in residues within a given conformational state by changing the protein’s conformational entropy rather than conformational changes of the whole protein. “Entropy driven allostery” or “dynamic allostery” was first described in 1951 for the cooperative binding of oxygen to haemoglobin. In the case of L-TGF-β1-GARP-avβ8 entropy of this complex decreases around avβ8 ligand binding site and increases at distant sites. This redistribution of conformational entropy (CER) leads to the repositioning of the various loops and accessibility of TGF-β1 to receptor binding (Fig. 1a).

Next, the authors revealed differences between L-TGF-β1 and L-TGF-β3, which might underly their distinct functional activities, i.e., in palatogenesis, a developmental process requiring TGF-β3 but not TGF-β1. Various amino acid loops, which differ in relative stability in fully latent L-TGF-β1 (stable) and partially latent L-TGF-β3 (flexible) are stabilised by binding to avβ8 and reduce local conformational entropy. When this entropy is spatially redistributed between these loops, flexibility is enhanced and causes release and, eventually, receptor exposure of mTGF-β (Fig. 1a). In case of L-TGF-β1, lower intrinsic entropy as compared to L-TGF-β3 results in less CER toward a specific domain, which despite insufficiency to release mTGF-β1, promotes exposure to TGF-βRII (Fig. 1a). Thus, dynamic allostery explains the mechanism of TGF-β3 latency/activation and functional dichotomy of TGF-β1 and 3.^1^ Notably, it remains unclear if L-TGF-β2 more closely resembles L-TGF-β1 or 3.

TGF-β is well known for its highly context and cell type-dependent effects, which constitute a major challenge for specific targeting in diverse diseases, particularly in cancer. The authors propose that this might be partly explained by CER, which determines (i) spatial and temporal aspects of TGF-β activation, (ii) the mode of signalling processing via aTGF-β or paracrine TGF-β (pTGF-β), and (iii) primary direction of TGF-β signalling to TGF-β-presenting or integrin-expressing cells, and nearby or remote cells. Consequently, authors speculate that targeting TGF-β activation through CER offers opportunities for directional context-dependent TGF-β activation, e.g., through basal entropy, entropic redistribution, or release. However, it remains to be determined how targeting CER will affect different pathologies. Nevertheless, results provide a structural basis for developing novel therapeutic approaches (Fig. 1b).

Further, the authors demonstrate that uncleaved TGF-β1 can induce sufficient TGF-β signalling to generate regulatory T cells (Tregs). Considering that GARP and integrin expression are not restricted to immune cells, mechanisms of L-TGF-β activation may not be limited to T cells and aTGF-β1 as well as pTGF-β1 do not necessarily be functionally equivalent. Indeed, we recently observed in pancreatic carcinoma cells that aTGF-β1 induces effects opposite to those of pTGF-β1 (the function of which can be mimicked in vitro by exogenously applied TGF-β1), i.e., *inhibition* of invasion and *stimulation* of growth.^3^ This suggests the possibility that unreleased aTGF-β1 acts as an endogenous inhibitor of released p/mTGF-β1 for specific cellular functions (Fig. 1b).

In non-immune, i.e., epithelial cells, L-TGF-β1 can be activated by agents other than avβ8 such as the secreted ECM protein, thrombospondin-1 (TSP-1) or reactive oxygen species (ROS) (Fig. 1b). Both are thought to induce a conformational rearrangement of LAP to prevent LAP from conferring latency on mTGF-β. It remains to be seen if TSP-1 or ROS, like avβ8, can enhance CER in L-TGF-β1 as seen with L-TGF-β1/GARP in Tregs and impair release of mTGF-β1. Since the authors focused on TGF-β activation in Tregs, which requires cell-cell rather than cell–matrix interactions, it remains unknown how LTBP may affect intrinsic flexibility or CER. This should be a topic for future studies since LTBPs play an important role in maintaining TGF-β latency by serving as chaperones to enhance the folding and secretion of pro-TGF-β.

Finally, authors propose that dynamic allostery, exemplified in the multicomponent avβ8/L-TGF-β1/3/GARP model system, is a general evolutionary strategy that controls the degree of latency of other TGF-β superfamily members. Besides TGF-β, myostatin (MSTN)/GDF-8 remains latent when complexed with its prodomain and unable to induce signalling without proteolytic activation. MSTN is a negative regulator of skeletal muscle growth that when overactivated can cause muscle atrophy, systemic inflammation and—along with TGF-β—cancer-associated cachexia. Notably, L-MSTN can act in a paracrine *and* autocrine fashion in vivo, possesses significant bioactivity and is able to bind to its receptor, ActRIIB.^4^ It will thus be exciting to measure its intrinsic entropy and compare it with that of L-TGF-β1 and 3.

From a therapeutic perspective, blocking release of p/mTGF-β1, or promoting the generation of aTGF-β1, by targeting L-TGF-β1 through modulation of CER could be exploited to interfere with epithelial–mesenchymal transition, a developmental programme employed by TGF-β1 to promote carcinoma progression^5^ (Fig. 1b), while analogous targeting of L-MSTN may cure cancer-associated cachexia. In conclusion, this paper represents a significant advancement in the understanding of TGF-β activation by unravelling a novel regulatory mechanism for tight regulation of protein function.
